# The Role of Session Zero in Successful Completion of Chronic Disease Self-Management Program Workshops

**DOI:** 10.3389/fpubh.2014.00205

**Published:** 2015-04-27

**Authors:** Luohua Jiang, Matthew Lee Smith, Shuai Chen, SangNam Ahn, Kristie P. Kulinski, Kate Lorig, Marcia G. Ory

**Affiliations:** ^1^Department of Epidemiology and Biostatistics, School of Public Health, Texas A&M Health Science Center, College Station, TX, USA; ^2^Department of Health Promotion and Behavior, College of Public Health, The University of Georgia, Athens, GA, USA; ^3^Department of Statistics, Texas A&M University, College Station, TX, USA; ^4^Division of Health Systems Management and Policy, School of Public Health, The University of Memphis, Memphis, TN, USA; ^5^Department of Health Promotion and Community Health Sciences, School of Public Health, Texas A&M Health Science Center, College Station, TX, USA; ^6^National Council on Aging, Washington, DC, USA; ^7^Stanford Patient Education Research Center, Department of Medicine, Stanford School of Medicine, Palo Alto, CA, USA

**Keywords:** attrition, retention, orientation session, evidence-based programs, chronic disease self-management program

## Abstract

**Background:** The Chronic Disease Self-Management Program (CDSMP) has been widely disseminated among various racial and ethnic populations. In addition to the six required CDSMP workshop sessions, the delivery sites have the option to offer a Session Zero (or zero class), an information session offered prior to Session One as a marketing tool. Despite assumptions that a zero class is helpful, little is known about the prevalence of these additional sessions or their impact on retaining participants in CDSMP workshops. This study aims to describe the proportion of CDSMP workshops that offered Session Zero and examine the association between Session Zero and workshop completion rates.

**Methods:** Data were analyzed from 80,987 middle-aged and older adults collected during a two-year national dissemination of CDSMP. Generalized estimating equation regression analyses were conducted to assess the association between Session Zero and successful workshop completion (attending four or more of the six workshop sessions).

**Results:** On average, 21.04% of the participants attended workshops that offered Session Zero, and 75.33% successfully completed the CDSMP workshop. The participants of the workshops that offered Session Zero had significantly higher odds of completing CDSMP workshops than those who were not offered Session Zero (OR = 1.099, *P* = <0.001) after controlling for participants’ demographic characteristics, race, ethnicity, living status, household income, number of chronic conditions, and workshop delivery type.

**Conclusion:** As one of the first studies reporting the importance of an orientation session for participant retention in chronic disease management intervention projects, our findings suggest offering an orientation session may increase participant retention in similar translational efforts.

## Introduction

In recent years, an increasing number of interventions have been deemed highly efficacious in the prevention and management of chronic diseases in randomized clinical trials (1). To disseminate the findings of those clinical trials, the critical next step is to examine whether the research-based studies can be translated into effective community-based programs that can recruit and retain large numbers of participants with various chronic diseases. Program retention is often a challenge in controlled clinical studies (2), but it can be even more pronounced in large-scale implementation efforts of community-based interventions (3–5). Participant attrition not only threatens the internal validity and statistical power of a project, but also compromises the intervention benefits received by participants because of the lack of adequate intervention dose (6, 7). Less rigorously controlled than clinical trials, translational efforts pose special challenges for participant engagement. For example, grand-scale translational intervention efforts typically allocate fewer resources to intensively track and follow-up with participants over time, which may impact retention success.

To address problems associated with participant attrition, a wide range of studies have investigated factors related to retaining participants in clinical trials and observational studies (8–15). A previous meta-analysis identified 12 basic themes for successful retention in longitudinal studies, which include community involvement, contact and scheduling methods, and financial incentives (16). To date, however, the strategies for successful retention in translational initiatives remain underexplored (17, 18).

The Chronic Disease Self-Management Program (CDSMP) has been introduced and widely disseminated into US communities as a method to empower patients to deal with their chronic conditions by enhancing their self-management skills (19). Drawing upon Social Learning Theory (20), CDSMP is an evidence-based, peer-led intervention consisting of six highly participative classes held for 2.5 h each, once a week, for six consecutive weeks (19). In addition to the six workshop sessions, some delivery sites are offering a Session Zero (or zero class), an information session offered prior to Session One as a marketing tool (21). The primary purpose of Session Zero is to provide an overview of the workshop, explain expectations for workshop participation, and confirm commitment of those who are interested in or have already registered for a workshop. This additional session also serves as an opportunity to collect baseline data from participants to alleviate administrative burden on workshop instructors and ensure time is not taken away from Session One of the workshop. Although designed as a recruitment tool, we believe that incorporating a Session Zero to CDSMP workshops may boost participant retention rates because those who were not firmly committed to the workshop might decide to opt out of the program at this time. The goals of the current study are to (1) describe the proportion of CDSMP workshops that offered Session Zero and (2) examine the association between Session Zero and workshop completion rates.

## Materials and Methods

### Data source and study population

Data for this study were obtained from a two-year nationwide delivery of CDSMP as part of the American Recovery and Reinvestment Act of 2009 (i.e., Recovery Act) Communities Putting Prevention to Work: Chronic Disease Self-Management Program Initiative (22). The U.S. Administration on Aging led this initiative in collaboration with the Centers for Disease Control and Prevention (CDC) and the Centers for Medicare and Medicaid Services (CMS) to support the translation of CDSMP in 45 states, Puerto Rico, and the District of Columbia (23). This initiative was executed between 2010 and 2012 to embed CDSMP delivery structures into statewide systems (22). Within the first two years of this initiative, more than 100,000 adults participated in 9305 workshops in 1234 U.S. counties (22). For this study, administrative records were utilized to determine whether or not a Session Zero was held. Data were analyzed from 80,987 participants aged 50 years or older whose programmatic records contained data about Session Zero attendance.

## Measures

### Dependent variable

CDSMP workshop attendance was the dependent variable for this study. As defined by the program developers and used in a variety of studies (24, 25), successful completion was defined as when CDSMP participants attended four or more of the six workshop sessions (22, 26), excluding Session Zero.

### Independent variables

Whether or not a workshop offered a Session Zero was recorded administratively and included in the database along with workshop attendance. Participants’ actual attendance of a Session Zero was not recorded. If offered, the Session Zero was usually offered 1–4 weeks prior to the workshop and targeted those who had already registered or who might have shown an interest in the workshop. This orientation session was also used to recruit acquaintances and/or family members of those who already registered for the workshop. The specific content of Session Zero varied by site; however, all of them should have provided an overview of the CDSMP workshop and its expectations for participation. Session Zero may also be used to collect baseline data to reduce interference with Session One of the workshop.

Workshop delivery sites included area agencies on aging (AAAs), healthcare organizations, residential facilities, community or multipurpose centers, faith-based organizations, educational institution, county health department, tribal center, workplace, and other (e.g., recreational center).

Socio-demographic factors included age (in years), sex (male vs. female), median household income (in $10,000 units), and living arrangement (living with others vs. living alone). Participants’ health status was measured by their number of self-reported chronic conditions (i.e., arthritis, cancer, depression, diabetes, heart disease, hypertension, lung disease, stroke, osteoporosis, and other chronic conditions).

### Statistical analysis

To compare the characteristics of the participants who attended workshops with a Session Zero and those who attended workshops without a Session Zero, we used χ*^2^* tests for categorical variables and two-sample *t*-tests for continuous variables. Because the participants were nested in workshops, generalized estimating equation (GEE) regression models were employed to investigate the association between successful workshop completion and Session Zero attendance. Specifically, the dependent variable of these regression models was successful workshop completion, while the independent variables were participant-level demographic and health characteristics. Furthermore, delivery site type was also included as an independent variable in the second GEE regression model. All the models included an exchangeable working covariance to account for the intraclass correlation among participants from the same workshop. Because the dependent variable is a binary variable; GEE analyses were conducted using SAS GENMOD procedure with a logit link function (SAS 9.3, SAS Institute, Inc., Cary, NC, USA).

## Results

Table 1 shows the proportions of participants who attended Session Zero and the workshop completion rates among the 10 types of delivery sites. Overall, 21.04% of the participants attended workshops with a Session Zero and 75.33% of participants successfully completed the CDSMP workshop. Among the 10 different types of delivery sites, the largest proportion of participants attending workshops with a Session Zero were at residential facilities (26.27%), while the smallest proportion of participants attending workshops with a Session Zero were at tribal centers (9.76%). With respect to workshop completion rates, workplaces had the highest completion rate (82.44%) and tribal center had the lowest completion rate (69.76%).

**Table 1:** **Session Zero attendance and CDSMP workshop completion rates by delivery site type**.

Workshop delivery site	Total, *N* (%)	Attended	CDSMP
		workshops	completion
		with a Session	(%)
		Zero (%)	
Senior Center/AAA	24,653 (30.44)	25.81	77.27
Health care organization	15,026 (18.55)	10.71	72.53
Residential facility	14,439 (17.83)	26.27	70.03
Community/multipurpose	8303 (10.25)	21.63	75.74
Faith-based organization	7127 (8.80)	22.25	78.88
Educational institution	1844 (2.28)	17.35	77.77
County health department	1013 (1.25)	19.64	69.89
Tribal center	205 (0.25)	9.76	69.76
Workplace	410 (0.51)	18.05	82.44
Other	7967 (9.84)	16.08	80.48
*Total*	80,987 (100.00)	21.04	75.33

*CDSMP, chronic disease self-management program; AAA, area agency on aging*.

As presented in Table 2, CDSMP participants who attended workshops with a Session Zero had significantly higher workshop completion rate than those who attended workshops without a Session Zero (77.85% vs. 74.66%, *P* < 0.001). Participants who attended workshops with a Session Zero were more likely to be female, African American or other race group, Hispanic, and live alone. In terms of chronic conditions, they were more likely to have diabetes and hypertension, but less likely to have arthritis, cancer, depression, and lung disease. The average numbers of chronic conditions were not significantly different based on Session Zero status. Finally, the participants who attended workshops with a Session Zero were significantly older and had lower household incomes.

**Table 2:** **Baseline characteristics of CDSMP participants by Session Zero status**.

	Total (*n* = 80,987)	Attended workshops	Attended workshops	*P*
		without a Session	with a Session	
		Zero (*n* = 63,946)	Zero (*n* = 17,041)	
Workshop completion	61,007 (75.33%)	47,740 (74.66%)	13,267 (77.85%)	<0.001
Female	59,669 (78.17%)	46,571 (77.47%)	13,098 (80.77%)	<0.001
Race				<0.001
White	45,673 (65.10%)	37,430 (67.63%)	8243 (55.64%)	
African American	15,929 (22.70%)	11,438 (20.67%)	4491 (30.32%)	
Asian/Pacific Islander	3057 (4.36%)	2511 (4.54%)	546 (3.69%)	
American Indian/Alaskan Native	1156 (1.65%)	969 (1.75%)	187 (1.26%)	
Other	4344 (6.19%)	2997 (5.42%)	1347 (9.09%)	
Hispanic	10,771 (15.78%)	7328 (13.49%)	3443 (24.74%)	<0.001
Living alone	10,968 (13.55%)	7868 (12.32%)	3100 (18.19%)	<0.001
Chronic conditions
Arthritis	34,769 (42.93%)	27,672 (43.27%)	7097 (41.65%)	<0.001
Cancer	7585 (9.37%)	6135 (9.59%)	1450 (8.51%)	<0.001
Depression	16,729 (20.66%)	13,819 (21.61%)	2910 (17.08%)	<0.001
Diabetes	26,033 (32.14%)	19,453 (30.42%)	6580 (38.61%)	<0.001
Heart disease	13,480 (16.64%)	10,630 (16.62%)	2850 (16.72%)	0.753
Hypertension	36,531 (45.11%)	28,647 (44.80%)	7884 (46.26%)	<0.001
Lung disease	14,045 (17.34%)	11,231 (17.56%)	2814 (16.51%)	0.001
Stroke	4220 (5.21%)	3316 (5.19%)	904 (5.30%)	0.534
	Mean (±SD)	Mean (±SD)	Mean (±SD)	
Age	67.03 (±14.60)	66.58 (±14.79)	69.87 (±13.03)	<0.001
Median income	5.07 (±1.30)	5.02 (±1.27)	4.87 (±1.40)	<0.001
Number of chronic conditions	2.20 (±1.71)	2.29 (±1.71)	2.26 (±1.70)	0.060

*CDSMP, Chronic Disease Self-Management Program*.

Table 3 illustrates the results of GEE regressions for workshop completion. As seen in Model 1, the participants of the workshops that offered Session Zero had significantly higher odds of completing CDSMP workshops than those who participated the workshops that did not offer a Session Zero (odds ratio [OR] = 1.087, *P* = 0.003). In addition, the likelihood of completing CDSMP workshops was higher among older participants (OR = 1.002, *P* = 0.003), females (OR = 1.089, *P* < 0.001), African Americans (OR = 1.147, *P* = 0.007), Asians and Pacific Islanders (OR = 1.354, *P* < 0.001), and Hispanics (OR = 1.171, *P* < 0.001). Conversely, the likelihood of completing the workshop was lower among those with higher household incomes (OR = 0.979, *P* = 0.008).

**Table 3:** **Generalized estimating equation regression models for successful workshop completion**.

	Model 1		Model 2	
	OR (95% CI)	*P*	OR (95% CI)	*P*
Session Zero offered	1.087 (1.030, 1.147)	0.003	1.099 (1.041, 1.161)	<0.001
Age	1.002 (1.001, 1.004)	0.003	1.004 (1.002, 1.005)	<0.001
Female	1.089 (1.039, 1.141)	<0.001	1.105 (1.054, 1.158)	<0.001
Race				<0.001^a^
White	0.989 (0.904, 1.082)	0.805	0.986 (0.900, 1.079)	0.751
African American	1.147 (0.904, 1.082)	0.007	1.133 (1.025, 1.251)	0.014
Asian/Pacific Islander	1.354 (1.179, 1.554)	<0.001	1.293 (1.126, 1.485)	< 0.001
American Indian/Alaska Native	0.916 (0.771, 1.088)	0.318	0.908 (0.760, 1.084)	0.284
Other	Ref	NA	Ref	NA
Hispanic	1.171 (1.087,1.263)	<0.001	1.145 (1.063,1.236)	<0.001
Living Alone	1.084 (0.977, 1.202)	0.127	0.930 (0.838, 1.031)	0.168
Median Income	0.979 (0.963, 0.994)	0.008	0.988 (0.972, 1.004)	0.128
Number of chronic conditions	1.005 (0.993, 1.017)	0.455	1.010 (0.998, 1.023)	0.093
Workshop delivery site				<0.001^a^
Senior Center/AAA			Ref	NA
Health Care Organization			0.837 (0.785, 0.893)	<0.001
Residential Facility			0.696 (0.655, 0.738)	<0.001
Community/Multipurpose			0.901 (0.835, 0.971)	0.007
Faith-based organization			1.150 (1.057, 1.251)	0.001
Educational institution			1.003 (0.871, 1.155)	0.968
County health department			0.797 (0.666, 0.954)	0.013
Tribal center			0.815 (0.559, 1.187)	0.286
Workplace			1.627 (1.166, 2.271)	0.004
Other			1.260 (1.158, 1.370)	<0.001

*^a^Overall Type 3 *P* value*.

*AAA, area agency on aging*.

After adding types of delivery site into the GEE regression model (Model 2), we found participants of the workshops that offered Session Zero still had significantly higher odds of completing CDSMP workshops (OR = 1.099, *P* < 0.001). Furthermore, the average workshop completion rates were significantly different among different delivery site types (*P* < 0.001), with residential facility had the lowest likelihood of completing the workshop (OR = 0.696, *P* < 0.001) while workplace had the highest likelihood (OR = 1.627, *P* = 0.004).

## Discussion

The results of the current study show about one in five CDSMP workshops in this national initiative offered a Session Zero. Among the 10 delivery site types, senior centers/AAAs and residential facilities had the highest rates of offering a Session Zero, while tribal centers and healthcare organizations had the lowest rates. These differences might be related to variation in population subgroups served by each delivery site type (27, 28), as well as site staff availability and facility constraints (e.g., space, time, competing commitments).

As suggested in a review of lessons learned from the National Institute of Aging’s Behavior Change Consortia (21), this study also demonstrates that participants who were offered orientation sessoins were more likely to complete intervention protocols. This finding indicates offering a Session Zero may not only facilitate participant recruitment, but also increase participant retention in grand scale community-based program dissemination efforts. Participants attending workshops with a Session Zero before the formal start of the workshop might have developed more support for and positive views of the program because they were given an opportunity to better understand the purpose, content, and expectations of the workshop. Meanwhile, attending a Session Zero may have given individuals who were not fully committed to the program a chance to re-evaluate their intention and opt out of the program if they felt they were not completely ready for it or thought it might not be beneficial for their preferences/needs. Therefore, the functions of a Session Zero with respect to retention might be twofold: (1) to strengthen the commitment of the participants by providing relevant information in advance and (2) to serve as a screening tool to identify those who are truly interested in the program and ready to participate. Future studies are warranted to study the details of these two potential functions of Session Zero.

Our results regarding the relationships between participants’ demographic characteristics and retention are consistent with the existing literature (11–13, 29–32). Specifically, we found that older participants and females had higher workshop completion rates. The relationship between race/ethnicity and participant retention in previous studies are mixed, although most report minority populations were harder to retain (33), some have reported relatively lower attrition rates among Hispanic participants (8, 10, 34). Here, we found that African American, Asian Pacific Islanders, and Hispanic participants had higher likelihood of completing the workshop successfully. Differences in CDSMP workshop attendance rates by participant demographics may have been reflective of the type of delivery site at which workshops were attended. Other studies have shown that certain delivery sites attract and serve different community subgroups (22, 27, 28). For example, better workshop attendance among older participants may reflect that larger proportions of older participants attend CDSMP workshops at residential facilities (also with higher attendance rates).

The strengths of this study include a large sample size and diverse race and ethnicity representation included in the analysis. The large sample not only allowed us to have high power to detect relatively small differences and associations, but also implies potential good generalizability of our findings. Furthermore, the study sample included 16% Hispanics, 23% African Americans, 4% Asian and Pacific Islanders, and 2% American Indian and Alaskan Natives. The substantial diversity of the sample composition further supports the generalizability of these study results.

Despite the study’s evident strengths, our findings need to be carefully interpreted in light of a few limitations. First, while data were collected that indicated whether or not workshops offered a Session Zero, participant attendance in these zero class sessions was not recorded. This means that participant attendance in a Session Zero could not be directly linked to their workshop attendance data. Second, these results were based on observational data, which limit our ability to determine a causal relationship between Session Zero attendance and CDSMP completion. Third, the number of variables collected at baseline was relatively small; therefore, although we were able to control for several important participant characteristics in the regression analyses, the identified associations in this study may be confounded by other unmeasured variables. Last, we were not able to investigate the relationship between Session Zero attendance and changes in health- and healthcare-related outcomes among these CDSMP participants because outcome measures were not available in this data collection effort.

In summary, including a Session Zero when delivering CDSMP workshops may be an important strategy for participant retention. Our findings suggest hosting a Session Zero may have implications for workshop attendance in similar translational efforts involving evidence-based programs for older adults. Given potential challenges associated with retaining participants in grand scale community-based interventions, offering a Session Zero before the formal start of the intervention might represent a feasible and efficient two-prong approach to help with participant retention in future translational projects.

## Conflict of Interest Statement

The authors declare that the research was conducted in the absence of any commercial or financial relationships that could be construed as a potential conflict of interest.

This paper is included in the Research Topic, “Evidence-Based Programming for Older Adults.” This Research Topic received partial funding from multiple government and private organizations/agencies; however, the views, findings, and conclusions in these articles are those of the authors and do not necessarily represent the official position of these organizations/agencies. All papers published in the Research Topic received peer review from members of the Frontiers in Public Health (Public Health Education and Promotion section) panel of Review Editors. Because this Research Topic represents work closely associated with a nationwide evidence-based movement in the US, many of the authors and/or Review Editors may have worked together previously in some fashion. Review Editors were purposively selected based on their expertise with evaluation and/or evidence-based programming for older adults. Review Editors were independent of named authors on any given article published in this volume.
